# Guidelines: the do’s, don’ts and don’t knows of feedback for clinical education

**DOI:** 10.1007/s40037-015-0231-7

**Published:** 2015-11-30

**Authors:** Janet Lefroy, Chris Watling, Pim W. Teunissen, Paul Brand

**Affiliations:** 1 Keele University School of Medicine, Clinical Education Centre RSUH, ST4 6QG Staffordshire, UK; 2 Schulich School of Medicine and Dentistry, Western University, Ontario, Canada; 3 Maastricht University and VU University Medical Center, Amsterdam, The Netherlands; 4 Isala Klinieken, Zwolle, The Netherlands

**Keywords:** Formative assessment, Feedback, Workplace based assessment, Feedback relationship, Feedback culture

## Abstract

**Introduction:**

The guidelines offered in this paper aim to amalgamate the literature on formative feedback into practical Do’s, Don’ts and Don’t Knows for individual clinical supervisors and for the institutions that support clinical learning.

**Methods:**

The authors built consensus by an iterative process. Do’s and Don’ts were proposed based on authors’ individual teaching experience and awareness of the literature, and the amalgamated set of guidelines were then refined by all authors and the evidence was summarized for each guideline. Don’t Knows were identified as being important questions to this international group of educators which if answered would change practice. The criteria for inclusion of evidence for these guidelines were not those of a systematic review, so indicators of strength of these recommendations were developed which combine the evidence with the authors’ consensus.

**Results:**

A set of 32 Do and Don’t guidelines with the important Don’t Knows was compiled along with a summary of the evidence for each. These are divided into guidelines for the individual clinical supervisor giving feedback to their trainee (recommendations about both the process and the content of feedback) and guidelines for the learning culture (what elements of learning culture support the exchange of meaningful feedback, and what elements constrain it?)

**Conclusion:**

Feedback is not easy to get right, but it is essential to learning in medicine, and there is a wealth of evidence supporting the Do’s and warning against the Don’ts. Further research into the critical Don’t Knows of feedback is required. A new definition is offered: Helpful feedback is a supportive conversation that clarifies the trainee’s awareness of their developing competencies, enhances their self-efficacy for making progress, challenges them to set objectives for improvement, and facilitates their development of strategies to enable that improvement to occur.

**Electronic supplementary material:**

The online version of this article (doi: 10.1007/s40037-015-0231-7) contains supplementary material, which is available to authorized users.

**Do’s—**educational activity for which there is evidence of efficacy

**Don’ts—**educational activity for which there is evidence of no efficacy or of harms (negative effects)

**Don’t Knows—**educational activity for which there is no evidence of efficacy

**Table 1:** Summary of guidelines. For the individual clinical supervisor giving feedback

	**Do’s for the** *process* **of feedback**	**Strength of recommendation**
Guideline 1.	Do realize that feedback is not just one person providing information to another to help them improve. Feedback is part of a social interaction influenced by culture, values, expectations, personal histories, relationships, and power. Do treat feedback as a conversation rather than as a commodity	Strong
Guideline 2.	Do recognize that trainees must perceive feedback as credible in order for it to be influential. Credible feedback is well-informed, typically by direct observation of the task or event, and it comes from a trustworthy source. Make sure that you as supervisor set a good example as a credible role model	Moderate
Guideline 3.	Decide the timing of feedback depending on the competence level of the trainee and on the complexity of the task	Moderate
Guideline 4.	Do encourage trainees to look for feedback and use it to enhance their performance	Moderate
	**Do’s for the** *content* **of feedback**	
Guideline 5.	Do tailor bespoke feedback to the individual trainee. The trainee might benefit from:	Strong
	– Reinforcement of key points done well	
	– Identification of key points which might have been done better or omissions	
	– Working out strategies for improving the quality of their work	
	– An increased self-awareness	
Guideline 6.	Do give specific feedback, focused on how the task was done and how that type of task should/might be done	Strong
Guideline 7.	Do make sure to indicate whether feedback is about necessary improvement for minimally acceptable performance or whether it is a reflection on possible variations to build upon adequate performance	Tentative
	Consider offering grades as an element of formative feedback if it seems that receiving grades will enhance the seeking of strategies for improvement. Conversely, avoid giving grades to trainees who you suspect will stop trying to learn if they get a good enough grade and to those who will give up if they get a poor grade	
Guideline 8.	Do ensure that feedback is actionable, enabling the trainee to construct strategies for improvement. After discussing the trainee’s performance of a task, provide some guidance or ‘scaffolding’ to enable them to step beyond their current competence	Strong
Guideline 9.	Do attend to trainee motivation when discussing strategies for improvement	Moderate
Guideline 10.	Regardless of the specific approach to feedback that is used, do engage the trainee in a reflective conversation that marries their self-assessment with your observations and elaborations	Tentative
	Several approaches have been described in the literature (sandwich, Pendleton, reflective feedback conversation, agenda-led outcome-based analysis, feedforward), but no single approach has been established to be the most effective. Rather, the likely best approach varies according to the learner, the teacher-learner relationship, and the context	
	**Don’ts**	
Guideline 11.	Don’t assume that a single approach to feedback will be effective with all trainees or in all circumstances. As the players and the contexts change, so too does the most useful approach to feedback. Don't assume:	Moderate
	– You know what a trainee wants to learn	
	– You know why a trainee is struggling	
	– You know if or why a trainee wants feedback	
	– You know what information a trainee takes out of a situation or feedback conversation	
Guideline 12.	Don’t provide feedback without follow-up. Trainees are unlikely to be influenced by feedback that is not followed by an opportunity for them to demonstrate improving performance	Moderate
Guideline 13.	Don’t provide feedback that is poorly informed (or is based on hearsay); doing so diminishes the value that trainees assign to feedback in general	Moderate
Guideline 14.	Don’t underestimate the emotional impact of feedback that is perceived as negative. Emotional distress may be a barrier to acceptance and use of feedback	Moderate
Guideline 15.	Don’t give grades without explaining the criteria for allocation of grades and providing strategies for improvement	Moderate
	**Don’t knows**	
Guideline 16.	What determines the credibility of feedback?	
Guideline 17.	How much is the right amount of content when giving feedback?	
Guideline 18.	What determines the ‘open and safe interaction’ in the feedback conversation?	
Guideline 19.	What influences the trainee’s response? (constructive or destructive outcomes)	
Guideline 20.	Is overt comparison with peers—when made by the supervisor—helpful to the trainee? Indeed, is overt comparison with required performance standards helpful?	
Guideline 21.	Does a written summary of the feedback discussion enhance learning?	
(what elements of learning culture support the exchange of meaningful feedback, and what elements constrain it?)		
	**Do’s**	**Strength of recommendation**
Guideline 22.	Do have a systems approach, building feedback into the learning processes	Moderate
Guideline 23.	Do support the development of longitudinal, trusting supervisor-trainee relationships in medical training; influential feedback thrives in the context of trusting relationships	Moderate
Guideline 24.	Do use video review with feedback as a component of training	Tentative
Guideline 25.	Do promote communities of practice in clinical workplaces in which feedback is routine, regular and valued	Moderate
Guideline 26.	Make sure that those who have a formal role in a workplace’s educational system are aware of that role and understand what learners’ educational objectives should be	Moderate
Guideline 27.	Make sure that the team give feedback regularly, reflect on the practice of giving feedback, and follow refresher courses to maintain and improve competency in providing feedback	Moderate
	**Don’ts**	
Guideline 28.	Don’t rely exclusively on faculty development to improve the effectiveness of feedback.	Moderate
Guideline 29.	Don’t allow formal assessments of clinical skills, such as the mini-CEX, to be completed without observation and feedback	Moderate
	**Don’t knows**	
Guideline 30.	What are the vital components that ensure a constructive system of workplace learning that caters to trainees, workers, and the educational system? How can the institution nourish a climate which encourages the provision and seeking of feedback?	
Guideline 31.	Is it most effective to give feedback to individuals alone or in a group setting?	
Guideline 32.	Does the use of formative assessment outcomes for summative purposes (such as having supervisors provide formative feedback that at the end of a rotation is also used for a summative assessment) corrupt a well-intentioned educational system?	

## Introduction

Feedback is considered of utmost importance for learning. Despite the importance of feedback and the attention it has received in scholarly literature, effective feedback remains difficult to achieve within the context of clinical education. The guidelines offered in this paper aim to amalgamate the literature on formative feedback into practical Do’s, Don’ts and Don’t Knows. The guidelines relate to formative feedback (i.e. exchange of information with the intent to support development) in clinical education (medical students and doctors learning in the workplace), but are also relevant to formative feedback associated with a summative assessment.

We have not attempted a systematic review of the considerable and growing body of literature on feedback in medical education. Rather, we offer recommendations based on published evidence from scientific exploration of the feedback process, and on our combined experience and study in this area. Below, we list the *Do’s, Don’ts,* and *Don’t Knows*. In the supporting paper that follows we briefly articulate what we regard as the key evidence for each Do and Don’t we have listed. In the summary (Table 1) we indicate the strength of this evidence and therefore of our recommendation using the criteria outlined in Table 2.

**Table 2:** Criteria for strength of recommendation

Strong	A large and consistent body of evidence
Moderate	Solid empiric evidence from one or more papers plus the consensus of the authors
Tentative	Limited empiric evidence plus the consensus of the authors

It is not easy to know what feedback will be useful to a trainee. There is a recognized feedback gap (between feedback given and what is received by the trainee [1]). What this means for supervisors is that delivering feedback without first diagnosing our trainee’s need and receptiveness risks wasting effort. The impact of formative feedback will depend on the strength of the trainee’s desire to improve and their confidence in their ability to do so [2]. To some extent these are personality traits (innate or learned earlier in life) but they can change with the trainee’s situation and we need to know how to promote both.

The guidance we have compiled is intended not only for clinical supervisors, but also for learners and for the institutions that support clinical learning. We suspect that one of the reasons that the quality and quantity of feedback has not improved greatly despite all the years of scrutiny and the libraries of words written about it is that the focus has been largely on how supervisors as individuals should construct and deliver feedback, with considerably less attention directed to how learners receive and respond to feedback, and to how institutions can create a culture in which feedback works. Clinical tutors may not be averse to giving useful feedback, but they may operate in an environment that limits their opportunity to do so. Learners want feedback, but they may be motivated more by competition for status or fear of failure than by the desire to improve as a clinician. Overcoming barriers to meaningful feedback demands both individual and institutional efforts. We therefore include a set of Do’s and Don’ts regarding the learning culture which are directed primarily at institutions wishing to promote feedback, in addition to our guidelines for the individual supervisor. We hope that by setting out the known Do’s and Don’ts and by encouraging study of the many Don’t Knows about feedback within our complex systems of clinical coaching, we can provide direction for these important efforts.

### Terminology

The term ‘trainees’ is used for both undergraduate and postgraduate learners, but where the stage of training is thought to influence the giving or receiving of feedback this is specified.

## Foundation paper

### Methods and ‘way of working’

The authors built consensus by an iterative process. Following an initial discussion to agree on the scope of the guidance and the criteria for selection of guidelines (see below), each author independently listed their Do’s, Don’ts, and Don’t Knows. These were amalgamated by JL and discussed for clarification where there was obvious conflict of Do’s and Don’ts. A lead author was identified for each of the compiled list who would provide an initial outline of the evidence. The compiled table of Do’s, Don’ts, and Don’t Knows with supporting evidence was then circulated for all authors to add evidence and comments. Where we considered that evidence was still conflicting or there was not a clear consensus following consideration by all authors, items which had been thought clear Do’s or Don’ts were moved into the Don’t Know section. This process was repeated once more for final agreement and the strength of each recommendation was determined by consensus.

The criterion for identifying a Do or a Don’t was that it was considered important to us as medical educators with our individual teaching experience and awareness of the literature. We did not set out to perform a systematic review of the large and growing body of literature on feedback in medical education. The range of undergraduate to postgraduate education teaching and research experience we had across three countries’ health systems led us to believe that we could compile useful guidelines. The Don’t Knows were identified as being important questions to this international group of educators which if answered would change practice.

Since the criteria for inclusion of evidence for these guidelines were not those of a systematic review, we avoided using the ABC indicators of strength and devised our own indicators which combine the evidence with the authors’ consensus (see Table 2).

## Results

In the initial discussion of the scope of the guidelines, it became clear that while we could provide guidance to individual clinical supervisors wishing to give better feedback, the impact of that guidance would be limited if they were working within a system that didn’t actively promote feedback as a way of improving. We therefore determined to divide our guidance into that for the individual clinical supervisor giving feedback and for those in positions of influence over the feedback culture in training systems and workplaces of medical students and doctors.

Our initial list of Do’s, Don’ts, and Don’t Knows numbered 65. We reduced this to the 32 listed largely by amalgamation with only two being dropped as unimportant on group reflection.

Items which had been thought clear Do’s or Don’ts but after examining the conflicting evidence were moved into the Don’t Know section were: Is comparison with peers helpful? Is comparison with required performance standards helpful? Can the same people give summative and formative feedback? (item no.32).

Conflicts of individual authors’ Do’s and Don’ts arose over whether trainees benefit from receiving grades with formative feedback. The consensus was that this may be useful to some trainees and a tentative recommendation was included.

The background evidence to each guideline is described and referenced in the following paragraphs. Table 1 is annotated with our judgement on the strength of our recommendation based on that evidence.

## Background evidence to guidelines for the individual clinical supervisor giving feedback

### Do’s for the *process* of feedback

1. *Do realize that feedback is not just one person providing information to another to help them improve. Feedback is part of a social interaction influenced by culture, values, expectations, personal histories, relationships, and power. Do treat feedback as a conversation rather than as a commodity.*

In a review paper on the role of feedback in self-assessment, Sargeant et al. [3] described how feedback from medical colleagues is part of a social process in which information is used to construct an understanding of one’s own performance. Reconciling and assimilating negative feedback with views held by the individual was found to be influenced by social context. Watling et al. explored how different professions, i.e. music, teacher training, and medicine, deal with feedback. The differences between professions described in that study highlight the influence of social and cultural values on the role and impact of feedback [4]. Viewing feedback only as ‘specific information about the comparison between a trainee’s observed performance and a standard, given with the intent to improve the trainee’s performance’ [5] ignores the complex ways in which culture, values, expectations, personal histories, relationships, and power manifest themselves through feedback [6].

Guideline 2. *Do recognize that trainees must perceive feedback as credible in order for it to be influential. Credible feedback is well-informed, typically by direct observation of the task or event, and it comes from a trustworthy source. Make sure that you as supervisor set a good example as a credible role model*.

A number of qualitative studies have shown that learners value feedback that they deem to be credible, but may dismiss feedback that they perceive to lack credibility [7–10]. Feedback that is negative or corrective is especially likely to be subjected to an appraisal of its credibility before learners will accept or act upon it. The credibility of feedback is influenced by the credibility of the source, by the process by which the feedback was informed and created, and by the content and characteristics of the feedback itself [9].

Guideline 3. *Decide the timing of feedback depending on the competence level of the trainee and on the complexity of the task.*

Studies of learners’ perceptions of effective feedback have highlighted the importance of timeliness to learners’ acceptance and use of feedback [7, 11], confirming that the all-too-frequent practice, within medical training, of providing performance feedback long after the event is rarely perceived by learners as useful. Although there is general agreement that feedback should be ‘timely’, the concept of optimal timeliness appears to be a nuanced one. For example, for simulation training of procedural skills, terminal feedback (at the end of the task performance) may be superior to concurrent feedback (during the task performance) for enhancing learning [12].

Hattie and Timperley [13] provide evidence that different levels of feedback deserve different timing. Thus immediate error correction during task acquisition is more effective than delayed, whereas immediate correction when trying to build fluency will detract from the learning of automaticity which is a process and therefore better discussed after the event.

Feedback after an audit showing comparatively poor clinical performance was most effective if given more than once and in writing as well as verbally [14].

Guideline 4. *Do encourage trainees to look for feedback and use it to enhance their performance.*

Our trainees may approach feedback with trepidation about the harm it might do to their self-esteem [15]; they may desire to make a good impression on their trainer among others; they may also desire the information which feedback gives them about how to improve [16]. These are the complex and largely unconscious psychological influences on feedback-seeking [17].

Trainees may hesitate to seek feedback on the very occasions when they might benefit from it most: situations where their performance has fallen below the required standard. In light of evidence for a heightened impact of feedback in these circumstances, the need to support trainees to seek and use feedback is especially pressing [14].

Research in non-clinical higher education shows that learners ask for feedback more frequently and see more benefits than costs in it as it is perceived to contain more valuable information. This assessment made by the learner of the potential value of feedback information is influenced by goal orientation [18, 19]. Teunissen et al. showed that this relationship between goal orientation and increased frequency of feedback seeking also holds in a population of postgraduate medical trainees [20]. There are experimental studies showing that although goal orientation is a fairly stable concept, a learning goal orientation can be fostered [21]. Supervisors should therefore encourage a learning frame of mind—this makes trainees more likely to accept formative feedback [17, 21]. In practical terms, this will involve welcoming discussions of the need to improve, encouraging goal-setting and planning of learning [22].

### Do’s for the *content* of feedback

Guideline 5. *Do tailor bespoke feedback to the individual trainee. The trainee might benefit from:* ‘reinforcement of key points done well’; ‘identification of key points which might have been done better or omissions’; ‘working out strategies for improving the quality of their work’; ‘an increased self-awareness’

Feedback needs to be tailored to the trainee’s perceptions [2]. It is most effective if directed at unsatisfactory elements of performance and linked to specific learning aims [23]. The content of feedback should therefore arise from a diagnostic and supportive dialogue between supervisor and trainee [24].

Learners actively process some (but not all) of the information they get in feedback [25, 26]. Relevance and credibility are important parameters for learners to decide how to act on feedback [10]. Both appear to increase when feedback is tailored to an individual’s needs.

Guideline 6. *Do give specific feedback, focused on how the task was done and how that type of task should/might be done*.

That feedback should be specific seems self-evident, and advice to teachers on giving feedback almost universally endorses the provision of specific feedback. General information unrelated to the performance, comments about a good or poor performance or compliments are less effective than specific comments [27, 28]. Lack of specificity has repeatedly been identified as an all-too-common weakness of the feedback that is typically exchanged in medical training [29]. When, however, one looks for evidence that increasing feedback specificity leads to more effective learning, the waters become murkier. Goodman et al. [30], for example, showed that increasing the specificity of feedback benefits initial performance, but discourages exploration, potentially undermining the deeper learning required for independent performance.

Kluger and DeNisi’s feedback intervention theory, derived from their meta-analysis of over 130 studies of feedback interventions in various settings, also posits that feedback becomes less effective as attention shifts away from the task and toward the individual; in short, feedback that is threatening to self-esteem is unlikely to be effective [28]. Sargeant invoked this theory to explain the difficulty practising physicians experienced in accepting and using negative or critical multisource feedback [31].

To sum up the advice from Hattie and Timperley [13] and Kluger and DeNisi [28], which is echoed by Archer [32] in his overview on the topic, feedback is most effective when directed at the task level and may assist in ‘deep processing and mastery of tasks’ when it is about processing of tasks or self-regulation. A ‘Don’t’ is providing feedback that focuses on the person level. According to Hattie and Timperley, person-oriented feedback ‘usually contains little task-related information and is rarely converted into more engagement, commitment to the learning goals, enhanced self-efficacy, or understanding about the task’ ([13], page 96).

Guideline 7. *Do make sure to indicate whether feedback is about necessary improvement for minimally acceptable performance or whether it is a reflection on possible variations to build upon adequate performance.*
  Consider offering grades as an element of formative feedback if it seems that receiving grades will enhance the seeking of strategies for improvement. Conversely, avoid giving grades to trainees who you suspect will stop trying to learn if they get a good enough grade and to those who will give up if they get a poor grade.

Self-regulation theories suggest that within each of our trainees are two basic self-regulation systems which co-exist but may conflict [33]. These two systems—the promotion (doing things because you want to) and prevention (doing things because you have to in order to avoid harm) approaches—may both be active in response to feedback [34]. It is important that the supervisor recognizes that his/her trainee is predominantly in promotion or prevention focus with respect to the focus of feedback, because positive feedback is more effective in motivating performance improvement for learners in promotion focus, while negative feedback is more useful in motivating performance improvement for learners in prevention focus [28]. Linking this with the evidence about goal orientation in Guideline No. 4, the promotion system generates goals which are experienced as desire for gratification, so learning goals when achieved will excite an increased desire to learn. The prevention self-regulatory system may encourage learning for fear of failure but this will feel like a necessity and achievement will cause relaxation rather than a desire for further learning [34]. The prevention system is active in individuals with performance goals—aiming to prove that one is already adequately competent and avoiding criticism. Feedback works best for learning when the trainee has learning goals rather than performance goals [17, 35] so it is important that the feedback itself should not push the trainee towards performance goals.

Grades are a clear and non-nuanced form of feedback which can trigger both promotion and prevention responses in trainees [28]. If a trainee is keen to know where they are in the opinion of the supervisor, their reasons can be explored by a supportive supervisor who can encourage a learning approach, aiming for self-awareness of competency and prioritization of areas for improvement. Receiving grades in this frame of mind was found to enhance the seeking of strategies for improvement, especially if criteria for allocation of grades are understood [26, 36]. Harmful effects of grades have also been noted in some participants in school, higher education and medical education [13, 26, 34], suggesting that making grades optional in formative feedback may be wise, with trainee choice being respected but perhaps explored by supervisors.

Guideline 8. *Do ensure that feedback is actionable, enabling the trainee to construct strategies for improvement. After discussing the trainee’s performance of a task, provide some guidance or ‘scaffolding’ to enable them to step beyond their current competence.*

Sadler suggests that for information to become feedback, it must enable the learner to take action to remedy the gap between actual and desired performance [37]. Information about ‘what went wrong’ that fails to enable learner action ‘how you can improve’ is merely ‘dangling data’ that is unlikely to motivate learning. Research into learners’ experiences of feedback has highlighted the value placed on feedback that is actionable [38]. Actionable feedback contains a roadmap for learner development; it provides explicit suggestions for building on strengths or addressing weaknesses in performance.

The theoretical concept of ‘scaffolding’ by tutors has been well developed by Wood et al. in their constructivist model of learning [39]. They based this on Vygotsky’s many studies in children of how the learner is helped to develop into their ‘zone of proximal development’ (beyond their current ability) by social interaction with tutors or peers [40]. In the social interactions of adult learning the scaffolding concept can also be helpful [27, 41–43].

The tasks of scaffolding as described by Wood et al. are:

- Orient the learner to the task
- Simplify into steps
- Motivate to maintain effort to achieve the goal
- Highlight critical features of the task
- Control frustration and the risk of failure
- Provide a model of the required actions

For trainees with a low level of competence, scaffolding involves giving directive feedback or specific instructions; for trainees with a high level of competence scaffolding can be less directive i.e. suggestions, hints and tips for (further) improvement (facilitative feedback) [27].

Guideline 9. *Do attend to trainee motivation when discussing strategies for improvement.*

In studies of educational psychology in children, motivation was a separate facet of the scaffolding of a challenging task [39]. Learning takes place at the edge of the comfort zone [40]. To prevent a child from giving up their efforts the teacher needs to encourage the child to believe that mastering the task is both possible and important. In adult learners, motivation is more likely to be internally generated [44] but it is no less important to learning, and is influenced by feedback [28, 34, 45]. In aiming for sufficient motivation to learn to do the task and sufficient self-efficacy that their effort is likely to succeed, clinical supervisors should check trainee response to their feedback as they go along. Trainee response depends on perceptions of the advice—does it challenge their way of doing things? (I need to change) and is the emotional impact of feedback positive? (I want to change and believe I can change). The trainee who will pay attention to the formative advice in feedback is the one who thinks they need to and can improve. It may be that the trainee had not identified the need for improvement before they got feedback from a credible source which alerts them to the need. The question then is whether they acknowledge that need and seem to want to improve. Clinical tutors can enhance motivation by making the suggestions in the feedback align with the trainee’s goals and therefore seem relevant [27].

Guideline 10. *Regardless of the specific approach to feedback that is used, do engage the trainee in a reflective conversation that marries their self-assessment with your observations and elaborations.*

By involving trainees in a discussion, supervisors can raise their awareness of their performance relative to their goals of quality performance through reflection-in-action and reflection-on-action [22, 32]. Coaching then includes confirming or challenging the trainee’s self-assessment, while recognizing that a challenge to the self-assessment of a junior learner whose understanding of the task is still superficial should differ from the challenge made to a more experienced trainee. Junior learners being less familiar with quality performance will rely more on the opinions of others (supervisors, peers) to make their self-assessment, and may need to be allowed an inflated self-efficacy and to receive the challenge step by step in order to keep trying. There are many factors which influence the effect of feedback, and the choice of how to deliver the feedback will depend on the task, the recipient and the feedback relationship [23]. Feedback should be ‘A supported sequential process rather than a series of unrelated events’ [32].

Several approaches to feedback have been described in the literature (sandwich, Pendleton, reflective feedback conversation, agenda-led outcome-based analysis, feedforward), but no single approach has been established to be the most effective. Rather, the likely best approach varies according to the learner, the teacher-learner relationship, and the context. The approaches mentioned are:

The feedback sandwich (in which the supervisor describes what went well, what can be improved, then re-emphasizes what went well) [46] harnesses the psychological effect of praise to enable the reception of criticism. This approach is thought helpful especially in the delicate start of a feedback relationship, but unnecessary once the relationship is robust. Evidence of its effectiveness is lacking.

Pendleton [47] outlined a method for giving feedback aiming to engage the learner in self-reflection and to balance positive and critical feedback. He suggested a series of ‘rules’:

- Check the learner wants and is ready for feedback.
- Let the learner give comments/background to the material that is being assessed.
- The learner states what was done well.
- The observer(s) state(s) what was done well.
- The learner states what could be improved.
- The observer(s) state(s) how it could be improved.
- An action plan for improvement is made.

The rules are intended to promote a safe and supportive environment, to encourage and incorporate self-assessment, and to generate recommendations rather than criticisms. The rules have been criticized as clunky and formulaic, but the framework can be helpful for learning to give and receive feedback.

Cantillon and Sargeant’s concept of the ‘reflective feedback conversation’ [48] is grounded in empiric work on the role of reflection as a critical link between receiving and using feedback.

The reflective feedback conversation unfolds like this:

- The teacher asks the learner to share concerns about performance.
- The learner describes concerns and what they would have liked to have done better.
- The teacher provides views and offers support, then asks the learner what might improve the situation.
- The learner responds, then the teacher elaborates on that response, correcting if needed, and checking understanding.

This approach focuses on the essential goals of feedback, encouraging learners to reflect, and motivating subsequent performance improvement. Importantly, the conversation should be viewed as a process rather than an event; revisiting and follow-up are often required.

Agenda-led outcome-based analysis (starts with the trainee’s agenda, looks at the outcomes they were aiming for, encourages self-assessment and problem-solving, provides balanced feedback and suggests alternatives). This method is described in Kurtz, Silverman and Draper’s Calgary Cambridge method for teaching communication skills [49] and is a learner-centred way of identifying the most helpful focus for a feedback discussion.

By contrast, the feedforward interview [34] is not actually a technique for feedback. It aims to avoid creating a discrepancy between a preferred standard and the actual state of affairs (seen as a key element of feedback, but also recognized as problematic for trainees who have low self-esteem) by focusing learners on their best performances. The trainee recalls peak moments in his/her performance and is asked to reflect on what conditions in themselves and their surroundings made that possible, then considers strategies to ensure sustainable peak performance. Kluger and van Dijk recommend periodical feedforward interviews with trainees about their peak experiences, partly in order to prepare the ground for necessary feedback to be received with a ‘promotion’ approach.

Don’t assume

Guideline 11. *Don’t assume that a single approach to feedback will be effective with all trainees or in all circumstances. As the players and the contexts change, so too does the most useful approach to feedback.*

- You know what a trainee wants to learn
- You know why a trainee is struggling
- You know if or why a trainee wants feedback
- You know what information a trainee takes out of a situation or feedback conversation

Individuals vary in their orientation toward clinical and educational tasks. Responses to feedback also differ between learners, even regarding similar performance on similar tasks. Dijksterhuis showed individual variability in the acceptance and responsiveness to feedback [50]. Kluger and van Dijk [34] proposed that regulatory focus theory might explain some of the observed variability in feedback responses, and Watling et al.’s naturalistic exploration of the usefulness of this theory showed it offered some insights into feedback responses in clinical learning situations [25].

Variability in the impact of feedback extends beyond the individual. Responses to feedback are also shaped by learning culture, and the norms and expectations it creates for feedback [38], And context, including the relational element of feedback, is increasingly recognized as influential; Telio [51] has recently highlighted the contextual influence of the ‘educational alliance’ that develops between teacher and learner on the feedback that is exchanged. In the face of such variability, teachers must develop versatile approaches to feedback that are grounded in an understanding of the learner. The feedback exchange is perhaps at its most effective when teachers’ and learners’ goals are aligned [38]. Alignment requires engagement and dialogue.

Guideline 12. *Don’t provide feedback without follow-up. Trainees are unlikely to be influenced by feedback that is not followed by an opportunity for them to demonstrate improving performance.*

Sargeant et al. [52] explored physicians’ reflective processes after they received multisource feedback. Reflection was found to influence not only the assessment and assimilation of feedback, but also the processing of their emotional responses to feedback. Furthermore, facilitated reflection was found to be useful in terms of enhancing the acceptance and use of feedback. The process of reflection, however, was often an extended one, especially when the feedback was perceived as negative or was in conflict with self-perception.

Guideline 13. *Don’t provide feedback that is poorly informed (or is based on hearsay); doing so diminishes the value that trainees assign to feedback in general.*

Surveys have demonstrated that trainees value feedback in principle, and value the provision of feedback as a desired quality of clinical teachers [53]. In reality, however, the quality of the feedback received in medical training is often reported as low, and poorly informed due to factors including limited direct observation of performance. As a consequence, trainees may begin to devalue external feedback in general, relying instead on self-assessment [10, 11]. It is encouraging that this need not be the case, and the quality of feedback improves after specific training of clinical faculty [29, 54, 55].

Guideline 14. *Don’t underestimate the emotional impact of feedback that is perceived as negative. Emotional distress may be a barrier to acceptance and use of feedback*.

Feedback intervention theory [28] posits that feedback which threatens self-esteem is much less likely to be effective. Sargeant provided a sobering example of this theory in action. In a study done two years after practising doctors received multisource feedback, she found that those who had received negative feedback that conflicted with their self-assessment experienced distressing and long-lasting emotions that limited their ability to accept and act upon the feedback [31]. Eva showed that the interpretation and acceptance of feedback was influenced by a complex interplay of emotions, including confidence and fear, and highlighted the importance of allowing the learner to maintain their self-concept when delivering feedback [2].

Guideline 15. *Don’t give grades without explaining the criteria for allocation of grades and providing strategies for improvement.*

The mini-CEX and other workplace assessments are most valuable as instruments for learning, rather than as a formal assessment of competence, but all too often grades are given with the comment boxes left blank [56]. In studies of the impact of grades in formative assessment, participants who reported that low grades motivated them to find strategies to improve did however need an explanation of the grade in order for it to be useful to them [26, 57]. Because of the potential for grades to demotivate or to reduce effort, it has been suggested that it might be wisest to avoid giving grades except when formally assessing the learner (in infrequent ‘high stakes’ assessments) [58].

### Don’t knows

Guideline 16. *What determines the credibility of feedback?*

Credibility is a fundamental determinant of the ultimate impact of feedback on a learner. How trainees make judgements about feedback’s credibility, and how well those judgements serve them educationally, deserve careful study [10].

Guideline 17. *How much is the right amount of content when giving feedback?*

How does the supervisor determine how many items of feedback are optimal (both strengths and weaknesses)? We do have some evidence from higher education studies which suggests that more is less, and that increasing complexity can even reduce the effect of feedback [27]. Recall of feedback is partial and selective [26]. According to cognitive load theory cognitive architecture leads to a working memory that is limited in its capacity when it has to deal with novel information [59]. A review on the cognitive load effects of visual and verbal instructions concluded that instructions that contain redundant information (for instance verbally stating what has already become visually obvious) more often inhibit than enhance learning [60].

A set of studies in various clinical training contexts could be helpful.

Guideline 18. *What determines the ‘open and safe interaction’ in the feedback conversation?*

Many, including Pendleton [47], have highlighted the importance of a safe and supportive climate for the exchange of feedback. But the specific constituents of a safe climate remain poorly understood, as are the ways in which individuals and organizations can promote it.

Guideline 19. *What influences the trainee’s response? (constructive or destructive outcomes)*

Regulatory focus theory may explain some of the individual variability in feedback responses [25, 34]. What we don’t know is how regulatory focus interacts with other influences on feedback’s impact, such as credibility. We also don’t know how regulatory focus can best be primed in order to enhance the impact of feedback.

How do the issues of vulnerability (self-efficacy), motivation to improve or to prevent harm, and credibility interact to give shape to constructive or destructive feedback in a workplace learning situation? How do we help trainees to believe that they can improve?

Responses to feedback are driven by individual traits and preferences *and* by values embedded within the learning culture. How these influences interact is inadequately understood, making it challenging to know where to focus our energies. Workplace learning theorists (e.g. Eraut [61], Billett [62]) have highlighted the need to understand how individual and the sociocultural influences on learning interact. Billett emphasizes the notions of affordances and agency; a learning environment offers a range of affordances, or opportunities to learn, but an individual learner must exercise agency to engage with those affordances. Feedback challenges may lie with either affordance (is good feedback made available to learners?) or agency (do learners choose to engage with feedback?), or both; the way these factors interact merits further study, as it has implications for where, and how, educators and institutions should channel their energies to improve feedback.

Guideline 20. *Is overt comparison with peers when made by the supervisor helpful to the trainee? Indeed, is overt comparison with required performance standards helpful?*

The evidence is rather conflicting on these two related questions, so although there is a lot of evidence we have decided that it may depend on the context and on what comparison is made.

Comparison with a standard of performance is part of one accepted definition of feedback in clinical education—‘specific information about the comparison between a trainee’s performance and a standard, given with the intent to improve the trainee’s performance’ [5]—but while this comparison must be going on in the mind of the feedback giver, it may or may not be helpful to the trainee receiving the feedback to be aware of their position relative to the standard.

According to Kluger and de Nisi’s meta-analysis, some feedback recipients feel content to be ‘good enough’ or become helpless when told they are not making the grade, to the detriment of their performance [28]. In the studies described, feedback is more likely to have a positive than a negative effect, but what we cannot be sure of as feedback providers is which of these is more likely in a given feedback situation, although there are predictive factors [23]. In a competency-based programme such as medical training it seems logical to reference the feedback given to required standards of competence. Trainees are anxious to know whether they are ‘making the grade’. Enabling support of learners to self-monitor in relation to competency requirements is an important goal [63, 64] and may be seen as such by our trainees which might explain why they desire and value grades. But do comparisons with standards help them to improve, or is it better for each trainee to strive for personal excellence? How can we determine which learners in which circumstances will find comparisons motivating, as opposed to disheartening?

What about comparison with peers? There is evidence that feedback becomes less effective as its focus moves away from the task and toward the self [13, 28]. Both self-referenced and other-referenced feedback (in)directly focus the attention to the self. Unfavourable comparisons with others may threaten self-esteem and promote a performance goal orientation, potentially hindering learning [17]. But despite these concerns, some research has suggested value in comparisons: one group showed that undergraduate medical students can be motivated by and can learn from self-comparison with peers [65].

This leads us to question does the feedback sign affect trainee clinicians learning of clinical skills in the same ways as it does psychology students’ performance writing essays [57], or is this effect context-dependent?

Eventually it boils down to the way the trainee’s psychology is affected. Do they feel they need to change? Want to change? Know how to change? The way these desires and understandings are shaped is an area of study which is still producing conflicting results so deserves further careful study.

Guideline 21. *Does a written summary of the feedback discussion enhance learning?*

Medical students have been found to value informal verbal feedback more than formal workplace-based assessment (WBA) with written feedback [66, 67]. One explanation is that feedback works best soon after the event, especially for a complex task such as consulting with a patient [11, 13]. The value of the written summary is therefore secondary but could include:

- Aiding reflection on the feedback at a later date
- Aiding discussion between tutor and trainee at a later date
- Enhancing tutor effort at the time of generating the feedback

The optimal role for written feedback represents an area for study.

*Guidelines for the learning culture:* what elements of learning culture support the exchange of meaningful feedback, and what elements constrain it?

### Do's

Guideline 22. *Do have a systems approach, building feedback into the learning processes.*

Institutions can create opportunities for longitudinal teacher-learner relationships to flourish, such as extended placements [68–70]. Supervision of a trainee can have built-in and protected routines of supervisor observation of trainee performance followed by feedback [32] and expectations of recurrent feedback following multiple assessment tasks over time [22]. Institutional expectations of supervision can include that written feedback is more than ticking boxes and ensure that the feedback instruments used enable specific explanations of the trainee’s position relative to required goals, and encourage the supervisor to suggest how to attain the goals [13, 71]. Expectations of the trainee might be reflection-on-feedback with some system of reinforcing implementation of strategies for improvement [32]. New trainees will require induction into the rules of the particular academic community.

In order to ensure a climate of feedback, an institution should provide a system of regular feedback not only for trainees but also for supervisors [32, 72].

In addition to providing faculty development courses, educational support can be offered to supervisors and the supervisors’ social networks can be used and supported to facilitate acceptance and use of feedback [73].

In the new movement towards programmatic assessment, progress and learning from feedback is emphasized and built into the system [74, 75]. This has been successful [76] although it has also met some difficulties in implementation [69], and when summative judgements are seen to be based on the formative assessments the feedback given may be less critical [77].

Some of these elements of a systems approach are further developed in the following guidelines.

Guideline 23. *Do support the development of longitudinal, trusting supervisor-trainee relationships in medical training; influential feedback thrives in the context of trusting relationships*.

When trainees can build a relationship with their supervisors, it allows them to trust the credibility of the feedback they receive and the alignment of the teacher’s goals with their own. As Bok et al. showed, durable teacher-learner relationships also prompt learners to seek feedback more readily [69].

Bates et al. [67] explored medical students’ perceptions of assessment and feedback in a longitudinal integrated clerkship—a setting that enables the development of durable, trusting, teacher-learner relationships. They found that such relationships afforded ‘constructive interpretation of critical feedback’ (p. 366); students were able to interpret even challenging or corrective feedback as supportive.

Within a trusting and supportive relationship, feedback is also more likely to be viewed as credible [50, 78]. Recognizing the centrality of relationship in the feedback process, the concept of the ‘educational alliance’ has been proposed as a framework for understanding the links between the teacher-learner relationship and the impact of the feedback generated within it [51, 79].

Guideline 24. *Do use video review with feedback as a component of training.*

The main advantage of video is that the trainee can review what they did and as well as getting feedback. The supervisor’s feedback may not differ whether following direct observation or following video observation but the trainee will be able to confirm the strengths and weaknesses in their own performance.

Supervisors differ considerably in the feedback they give after reviewing the same videotaped consultation [80]. This raises the question of whether the supervisor’s feedback adds value to the trainee self-assessing their own videoed consultation. In a systematic review Hammoud et al. concluded that video review with self-assessment alone was not found to be generally effective for medical students, but when linked with expert feedback it was superior to traditional feedback alone [81]. This is a strong argument in favour of building video review with feedback into educational programmes especially to address the important but less self-evident problems.

Potential disadvantages include the relative complexity of arranging filming and viewing and that if videos are being selectively proffered for feedback the trainee may choose their best performances.

Guideline 25. *Do promote communities of practice in clinical workplaces in which feedback is routine, regular and valued.*

This can be a helpful approach in turning the workplace into a powerful learning environment when it can otherwise be a frustratingly hard place to change [6]. If the people working together in a workplace realize that everyone is also a learner and that feedback is a powerful way of learning, an environment is created in which providing feedback is considered ‘normal’. This would mean, for example, that trainees are encouraged to give feedback to their supervisors [82, 83]. It has also been found in the training of athletes and musicians that critical feedback is exchanged more readily when it is normalized by a learning culture [38, 84]. These studies provide at least indirect support for the idea that when feedback becomes a routine part of a learning culture, it may be more readily taken up and used by learners. And part of becoming ‘routine’ is that feedback, including critique, is exchanged very frequently.

Guideline 26. *Make sure that those who have a formal role in a workplace’s educational system are aware of that role and understand what learners’ educational objectives should be*.

In a study of residents’ expectations of their clinical teachers, Boor et al. found that, next to the importance of a good relationship, learners value clinical supervisors who are aware of the educational system and expectations and who can apply that knowledge to the individual learner [85]. Van der Vleuten’s comments on programmatic assessment are useful here: ‘If a programme of assessment is to provide meaningful outcomes, all the players should understand what they are doing, why they are doing it, and why they are doing it *this way*.’ [86] If we substitute ‘feedback’ for ‘assessment’, the comment rings equally true.

Guideline 27. *Make sure that the team give feedback regularly, reflect on the practice of giving feedback, and follow refresher courses to maintain and improve competency in providing feedback*.

Lack of faculty insight in the assessment process remains an issue [87]. The feedback landscape described by Evans [1] indicates the need for tutor training: the tutor must accurately diagnose academic and social needs; understand and empathize with the learner’s perspective, and have skills to employ appropriate scaffolding tools. Although no one technique of giving feedback has proven superiority and different individual trainees may respond to different approaches, there is evidence that it has been helpful to train supervisors in techniques of providing feedback constructively, and their behaviour changes in providing more useful feedback [73].

### Don'ts

Guideline 28. *Don’t rely exclusively on faculty development to improve the effectiveness of feedback*.

Historically, faculty development in feedback delivery has been the primary approach to improving the quality and effectiveness of feedback [29, 73]. This focus on how feedback is given ignores the important element of how it is received by learners [88]. The crucial role of learning culture in making effective feedback possible, normalizing constructive criticism, and establishing the value of feedback for learning is also missed by an approach focused on individual teachers [38]. Faculty development is important but not sufficient; attention must also be paid to learners’ receptivity to feedback and to the elements of the learning culture that support or constrain the feedback exchange.

Guideline 29. *Don’t allow formal assessments of clinical skills, such as the mini-CEX, to be completed without observation and feedback.*

Although designed to rely on observation of at least one clinical encounter and including space for documentation of feedback discussions, paper instruments such as the mini-CEX are frequently used as tick-box exercises to enable progression of trainees [56].

### Don’t knows

Guideline 30. *What are the vital components that ensure a constructive system of workplace learning that caters to trainees, workers, and the educational system? How can the institution nourish a climate which encourages the provision and seeking of feedback?*

Although we found several Do’s relating to the system approach to learning in the workplace, these feedback approaches are largely limited to individuals, despite ways of working in health care that increasingly demand competent team function. There are few studies on the impact of the provision of feedback to teams of individuals and the outcomes are variable, as described in a review by Gabelica et al. [89]. They raised an interesting paradox: ‘On the one hand… feedback might impact a huge diversity of critical team processes (amongst which the three most frequent variables: motivation, team goal, and collaboration/cooperation) and emergent states (among which the most frequent variables: collective efficacy, cohesion, outcome expectations, and task concern/interest) and occasionally have a direct effect on team performance (in 23 studies overall). On the other hand, some studies confirmed that feedback might not always lead to significant or at least measurable changes and thus not fulfil its function as a leverage point that can be used to support teams.’ They conclude that the real question is not whether feedback works, but under what circumstances is works best. A model is provided that highlights key factors that might enhance and support feedback effectiveness. Feedback about and during the *process* of teamwork was more reliably effective than feedback about *performance* given to the team or to individuals within the team. They recommended further research into what makes for effective feedback about team processes—how teams communicate, interact, establish their team atmosphere, define team objectives and strategies, monitor performance, come to a common understanding of the task and its requirements, build on each other’s expertise, make team decisions and coordinate in an efficient way.

Guideline 31. *Is it most effective to give feedback to individuals alone or in a group setting?*

In group learning of clinical skills, feedback to the trainee(s) who have experimented with a task is generally given by and in front of the group. This can include group feedback on a videoed real patient consultation. The advantages to this approach are that a range of feedback perspectives are gained, feedback-giving is role-modelled, and observers learn vicariously. The disadvantages are reduced control over content and volume of feedback, plus the risk of a negative emotional impact. In situations where it might be possible to give feedback either individually or in a group setting, we do not know whether the advantages outweigh the disadvantages. There are studies which have found learner preferences for group feedback [90] and for individual feedback [1] and it is clear that the context matters [28].

Guideline 32. *Does the use of formative assessment outcomes for summative purposes (such as having supervisors provide formative feedback that at the end of a rotation is also used for a summative assessment) corrupt a well-intentioned educational system?*

Programmatic assessment (a system of frequent formative assessments also used for end-of-year summative judgements) is designed to optimize learning and reduce exam stress [86]. Evidence is now emerging from qualitative evaluations of programmatic assessment curricula which raises questions about the mixing of formative and summative assessment. A qualitative study with clinical undergraduate veterinary students and their supervisors highlighted that both struggled with formative assessments that are used as ‘data points’ for a final summative judgment. As a result, the formative assessments did not play the powerful assessment-for-learning role they are meant to have in a curriculum based on programmatic assessment [69].

Medical education not only blurs the line, at times, between summative and formative assessment, but also blurs the line for its teachers between the roles of coach and assessor. Although these roles are distinct—coaches provide formative feedback while assessors make summative judgements—the same teacher is routinely expected to play both roles simultaneously and for the same learner. Recent literature has begun to challenge this approach, suggesting that the quality and impact of feedback may be compromised when the teacher is assigned this dual role [38, 91, 92]. Exactly how feedback is impacted by this practice, and whether feedback would be more effective if the coaching and assessment roles were separated, remains unknown.

## Summary

We have produced what we hope is a usable set of guidelines in an area that is central to teaching. Our work adds to the literature by interpreting a diverse and sometimes contradictory range of research and opinion for the clinical supervisor and his/her manager.

**Fig. 1:**
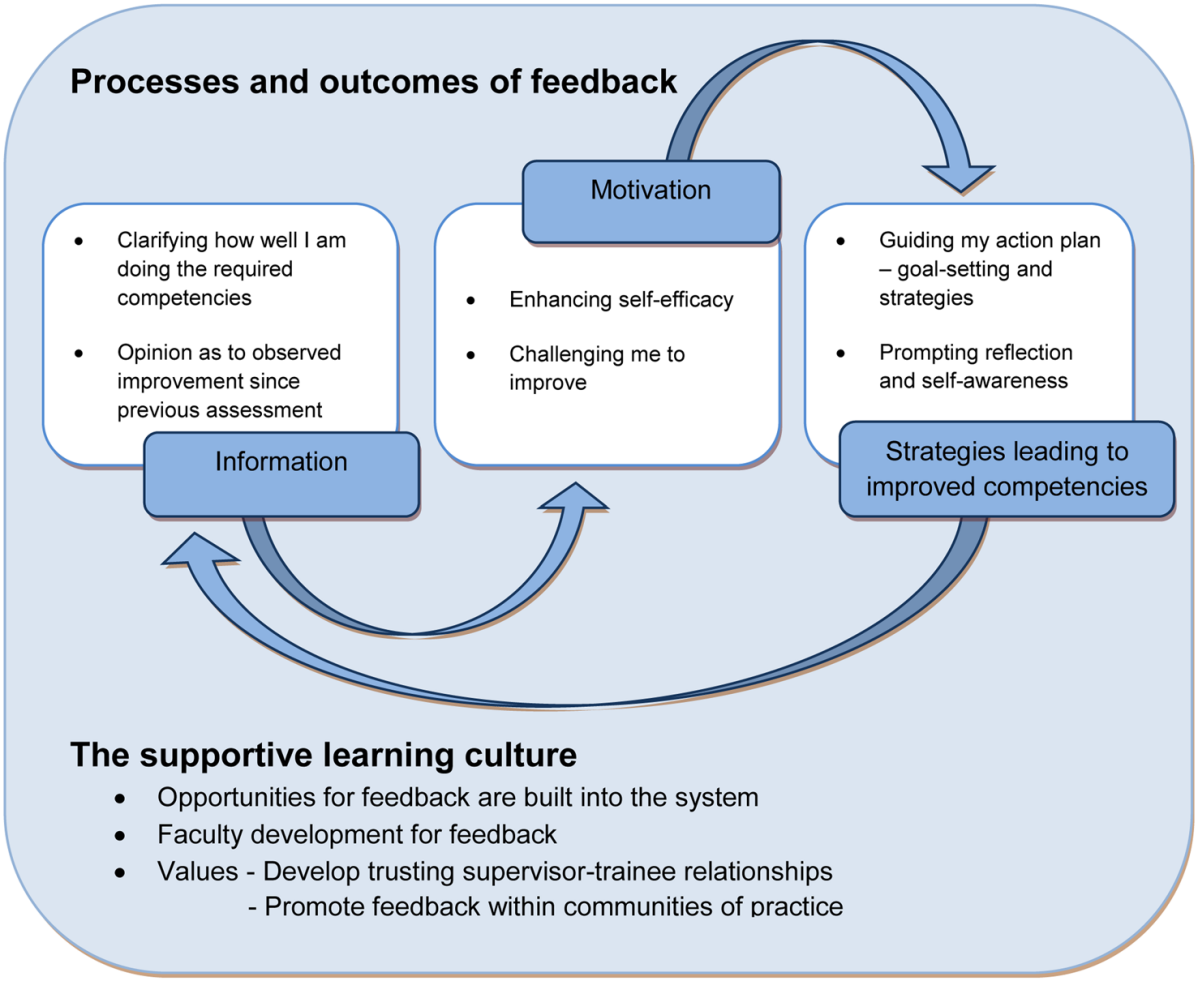
Feedback processes and outcomes—what the trainee wants from the feedback relationship

We have also developed a visual representation of the feedback process and outcomes (Fig. 1) which we offer as a summary of the guidelines from the viewpoint of the recipient of feedback. Trainees are looking for information about their performance and motivation to be/aim to be exemplary clinicians. The feedback process is incomplete if it does not result in the generation of strategies for improvement—either recommendations, or self-generated as a result of feedback. And the best feedback process loops back into a subsequent assessment with feedback about whether this has resulted in improved clinical performance. These processes and outcomes will flourish in the supportive learning culture of systematic dialogic feedback.

Our combination of perspectives and our iterative, consensus-building approach to creating these guidelines are strengths of this work. An obvious weakness is the lack of a systematic search method; as a consequence, we will have missed some useable evidence. We did, however, use the systematic reviews we know of to ensure that the evidence therein has contributed to these guidelines.

## Conclusion

Feedback resists one-size-fits-all guidelines. The wealth of research on feedback paints a picture of a nuanced process, with a great potential to help learners in all sorts of circumstances, but also a process that is fraught with variability and unpredictability, and influenced by individuals, contexts, and culture. In short, feedback is both an opportunity and a threat for teachers and learners. But we must not simply throw up our hands. Feedback may be complex, but it is essential to learning in medicine. We encourage supervisors to support best practices in feedback by embracing the Do’s we have identified and banishing the Don’ts. And we invite researchers to explore the intriguing and critical Don’t Knows of feedback, so that the field continues to advance and the next set of guidelines will be even more firmly grounded in empirical work. Our work has challenged us to reconsider the very definition of feedback in medical education. We offer a new definition that may help to shape future conversations*: Helpful feedback is a supportive conversation that clarifies the trainee’s awareness of their developing competencies, enhances their self-efficacy for making progress, challenges them to set objectives for improvement, and facilitates their development of strategies to enable that improvement to occur.*

## Electronic supplementary material

(DOCX 24 kb)
