# Supplementary material for: Guidelines: the do’s, don’ts and don’t knows of feedback for clinical education
Source: Perspect Med Educ. 2015 Nov 30;4(6):284–99. doi: 10.1007/s40037-015-0231-7 (PMC4673072; doi:10.1007/s40037-015-0231-7)
Supplement: Supplementary file 1 — (DOCX 24 kb) [file 40037_2015_231_MOESM1_ESM.docx]

**Do’s, don’ts and don’t knows of feedback for clinical education**

**Janet Lefroy · Chris Watling · Pim W. Teunissen · Paul Brand

Perspectives in Medical Education, DOI: 10.1007/s40037-015-0231-7**

***Helpful feedback*** **is a supportive conversation that clarifies the trainee’s awareness of their developing competencies, enhances their self-efficacy for making progress, challenges them to set objectives for improvement, and facilitates their development of strategies to enable that improvement to occur.**

| **Summary of guidelines**  **For the individual clinical supervisor giving feedback** | |
| --- | --- |
| **Do’s for the *process* of feedback** | **Strength** |
| 1. Do realize that feedback is not just one person providing information to another to help them improve. Feedback is part of a social interaction influenced by culture, values, expectations, personal histories, relationships, and power. Do treat feedback as a conversation rather than as a commodity | **Strong** |
| 2. Do recognize that trainees must perceive feedback as credible in order for it to be influential. Credible feedback is well-informed, typically by direct observation of the task or event, and it comes from a trustworthy source. Make sure that you as supervisor set a good example as a credible role model | **Moderate** |
| 3. Decide the timing of feedback depending on the competence level of the trainee and on the complexity of the task | **Moderate** |
| 4. Do encourage trainees to look for feedback and use it to enhance their performance | **Moderate** |
| **Do’s for the *content* of feedback** |  |
| 5. Do tailor bespoke feedback to the individual trainee. The trainee might benefit from:   - Reinforcement of key points done well - Identification of key points which might have been done better or omissions - Working out strategies for improving the quality of their work - An increased self-awareness | **Strong** |
| 6. Do give specific feedback, focused on how the task was done and how that type of task should/might be done | **Strong** |
| 7. Do make sure to indicate whether feedback is about necessary improvement for minimally acceptable performance or whether it is a reflection on possible variations to build upon adequate performance  Consider offering grades as an element of formative feedback if it seems that receiving grades will enhance the seeking of strategies for improvement. Conversely, avoid giving grades to trainees who you suspect will stop trying to learn if they get a good enough grade and to those who will give up if they get a poor grade | **Tentative** |
| 8. Do ensure that feedback is actionable, enabling the trainee to construct strategies for improvement. After discussing the trainee’s performance of a task, provide some guidance or ‘scaffolding’ to enable them to step beyond their current competence | **Strong** |
| 9. Do attend to trainee motivation when discussing strategies for improvement | **Moderate** |
| 10. Regardless of the specific approach to feedback that is used, do engage the trainee in a reflective conversation that marries their self-assessment with your observations and elaborations  Several approaches have been described in the literature (sandwich, Pendleton, reflective feedback conversation, agenda-led outcome-based analysis, feedforward), but no single approach has been established to be the most effective. Rather, the likely best approach varies according to the learner, the teacher-learner relationship, and the context | **Tentative** |
| **Don’ts** |  |
| 11. Don’t assume that a single approach to feedback will be effective with all trainees or in all circumstances. As the players and the contexts change, so too does the most useful approach to feedback. Don't assume:   - You know what a trainee wants to learn - You know why a trainee is struggling - You know if or why a trainee wants feedback - You know what information a trainee takes out of a situation or feedback conversation | **Moderate** |
| 12. Don’t provide feedback without follow-up. Trainees are unlikely to be influenced by feedback that is not followed by an opportunity for them to demonstrate improving performance | **Moderate** |
| 13. Don’t provide feedback that is poorly informed (or is based on hearsay); doing so diminishes the value that trainees assign to feedback in general | **Moderate** |
| 14. Don’t underestimate the emotional impact of feedback that is perceived as negative. Emotional distress may be a barrier to acceptance and use of feedback | **Moderate** |
| 15. Don’t give grades without explaining the criteria for allocation of grades and providing strategies for improvement | **Moderate** |
| **Don’t knows** |  |
| 16. What determines the credibility of feedback? | |
| 17. How much is the right amount of content when giving feedback? | |
| 18. What determines the ‘open and safe interaction’ in the feedback conversation? | |
| 19. What influences the trainee’s response? (constructive or destructive outcomes) | |
| 20. Is overt comparison with peers—when made by the supervisor—helpful to the trainee? Indeed, is overt comparison with required performance standards helpful? | |
| 21. Does a written summary of the feedback discussion enhance learning? | |

| **Guidelines for the learning culture** (what elements of learning culture support the exchange of meaningful feedback, and what elements constrain it?) | |
| --- | --- |
| **Do’s** | **Strength** |
| 22. Do have a systems approach, building feedback into the learning processes | **Moderate** |
| 23. Do support the development of longitudinal, trusting supervisor-trainee relationships in medical training; influential feedback thrives in the context of trusting relationships | **Moderate** |
| 24. Do use video review with feedback as a component of training | **Tentative** |
| 25. Do promote communities of practice in clinical workplaces in which feedback is routine, regular and valued | **Moderate** |
| 26. Make sure that those who have a formal role in a workplace’s educational system are aware of that role and understand what learners’ educational objectives should be | **Moderate** |
| 27. Make sure that the team give feedback regularly, reflect on the practice of giving feedback, and follow refresher courses to maintain and improve competency in providing feedback | **Moderate** |
| **Don’ts** |  |
| 28. Don’t rely exclusively on faculty development to improve the effectiveness of feedback | **Moderate** |
| 29. Don’t allow formal assessments of clinical skills, such as the mini-CEX, to be completed without observation and feedback | **Moderate** |
| **Don’t knows** |  |
| 30. What are the vital components that ensure a constructive system of workplace learning that caters to trainees, workers, and the educational system? How can the institution nourish a climate which encourages the provision and seeking of feedback? | |
| 31. Is it most effective to give feedback to individuals alone or in a group setting? | |
| 32. Does the use of formative assessment outcomes for summative purposes (such as having supervisors provide formative feedback that at the end of a rotation is also used for a summative assessment) corrupt a well-intentioned educational system? | |

**Strength of recommendation**

| **Strong** | **A large and consistent body of evidence** |
| --- | --- |
| **Moderate** | **Solid empiric evidence from one or more papers plus the consensus of the authors** |
| **Tentative** | **Limited empiric evidence plus the consensus of the authors** |
